# Fertility Preservation in Women with Endometriosis: Indications, Timing, Techniques, and Reproductive Outcomes

**DOI:** 10.3390/medicina62101899

**Published:** 2026-10-01

**Authors:** Luca Tramontano, Jean-Marie Wenger, Roberto Marci

**Affiliations:** 1 WENGER Center for Gynecology, Endometriosis and Fertility, Hôpital de La Tour, Rue de Veyrot 39, 1217 Meyrin, Switzerland; 2 Department of Translational Medicine and for Romagna, University of Ferrara, Via Luigi Borsari 46, 44121 Ferrara, Italy

**Keywords:** endometriosis, fertility preservation, oocyte cryopreservation, oocyte vitrification, ovarian endometrioma, ovarian reserve, ovarian surgery, live birth

## Abstract

Endometriosis is a chronic inflammatory disease frequently associated with infertility. Reproductive potential may be impaired by ovarian endometriomas, age-related decline, inflammatory and anatomical mechanisms, and ovarian surgery. This comprehensive narrative review summarises current evidence on fertility-preservation strategies in women with endometriosis, based on a structured literature search updated to 9 August 2026. Oocyte vitrification is the most frequently used and best-studied technique, while embryo cryopreservation may be considered according to individual circumstances, and ovarian tissue cryopreservation currently has a limited disease-specific role. Observational studies consistently show that increasing age and previous ovarian surgery are associated with fewer retrieved and mature oocytes, supporting counselling before ovarian surgery whenever feasible. Recent phenotype-stratified data further suggest that ovarian endometriosis is more consistently associated with reduced ovarian response than superficial or deep disease without ovarian involvement. Gonadotropin-releasing hormone antagonist and progestin-primed stimulation protocols both appear feasible, and repeated cycles may increase cumulative oocyte yield. Vitrified oocytes can survive warming and result in live birth; however, reproductive outcomes depend strongly on age and the number of oocytes available. Coexisting adenomyosis illustrates a limitation of gamete preservation because uterine factors may continue to affect implantation, miscarriage and later pregnancy outcomes. Recent follow-up data demonstrate that many women conceive spontaneously and that only a proportion return to use cryopreserved material. Consequently, the incremental benefit of preventive oocyte cryopreservation over expectant management and later fertility treatment remains unknown. Current evidence does not support systematic fertility preservation for all women with endometriosis. Early counselling and selective treatment should be considered, particularly in women with bilateral or recurrent ovarian disease, previous or planned ovarian surgery, and delayed reproductive plans.

## 1. Introduction

Endometriosis is a chronic inflammatory disorder affecting women throughout their reproductive years and is frequently associated with pelvic pain, impaired quality of life, and infertility [1,2]. Its effect on reproductive potential is multifactorial and may involve distorted pelvic anatomy, inflammation, altered follicular function, ovarian endometriomas, coexisting adenomyosis, and the consequences of ovarian surgery. These reproductive implications have become increasingly relevant as many women postpone childbearing and may therefore remain exposed for longer periods to age-related fertility decline, disease progression, and repeated therapeutic interventions.

Ovarian endometriosis represents a particular concern. Endometriomas may coexist with reduced ovarian reserve and lower response to controlled ovarian stimulation, while their surgical treatment may further compromise the functional ovarian cortex through inadvertent removal of healthy tissue, vascular injury, and haemostatic energy. This potential double effect of disease and treatment has led to increasing interest in fertility-preservation strategies, especially before ovarian surgery.

Oocyte vitrification is currently the most frequently used fertility-preservation technique in women with endometriosis. Embryo cryopreservation may represent an alternative for selected patients, whereas ovarian tissue cryopreservation has a much more limited and insufficiently defined role in this population. Nevertheless, the feasibility of retrieving and cryopreserving oocytes does not itself demonstrate that the procedure improves the probability of completing future family plans. Relevant outcomes include the number and maturity of retrieved oocytes, the proportion of women who subsequently return to use their material, oocyte survival after warming, pregnancy and live-birth rates, spontaneous conception after fertility preservation, and the incremental benefit compared with a conventional reproductive pathway.

The indication for fertility preservation in endometriosis has long been debated, with earlier reviews emphasising individualisation and the lack of evidence supporting universal treatment [3,4]. A 2025 systematic review subsequently identified only 10 observational studies and no randomised trials specifically evaluating oocyte cryopreservation in women with endometriosis. The included studies were heterogeneous in patient selection, disease phenotype, previous surgery, ovarian reserve, stimulation protocols, and reported outcomes. Increasing age and previous ovarian surgery were consistently associated with a lower oocyte yield, whereas adequate long-term follow-up on utilisation and live birth was largely absent [5].

More recent data have partly addressed this limitation. In a bicentric cohort with a median follow-up of 4.6 years, approximately 41% of women with available follow-up attempted conception; among them, 63.2% achieved at least one pregnancy and 40.8% achieved at least one live birth. Overall, 40 of 200 women (20%) had used their cryopreserved oocytes or embryos by the end of follow-up. Importantly, half of the pregnancies were conceived naturally, demonstrating that reproductive outcomes after fertility preservation cannot be attributed exclusively to the use of cryopreserved material [6].

Current recommendations therefore emphasise counselling rather than routine treatment. The 2022 ESHRE guideline recommends discussing the advantages and disadvantages of fertility preservation in women with extensive ovarian endometriosis, while explicitly acknowledging that its true benefit remains unknown and that evidence is insufficient to support systematic oocyte cryopreservation for all women with endometriosis [7].

This review summarises current evidence on fertility preservation in women with endometriosis, with particular attention to disease- and surgery-related reproductive impairment, available techniques, controlled ovarian stimulation, utilisation and reproductive outcomes, patient selection, optimal timing, counselling, and priorities for future research.

## 2. Literature Search Strategy

A structured literature search was conducted in PubMed/MEDLINE and supplemented by Europe PMC, ClinicalTrials.gov, reference-list screening, and targeted verification of relevant guidelines and primary studies. A recent systematic review had already searched PubMed, Embase, Web of Science, Scopus, and ClinicalTrials.gov from database inception to 31 December 2023 [5]. The present review therefore used that work as the systematic evidence base through 2023 and performed an update covering 1 January 2024 to 9 August 2026.

Search terms combined “endometriosis”, “ovarian endometriosis”, and “endometrioma” with “fertility preservation”, “oocyte cryopreservation”, “oocyte vitrification”, “embryo cryopreservation”, “ovarian tissue cryopreservation”, “ovarian stimulation”, “progestin-primed ovarian stimulation”, “oocyte retrieval”, “ovarian surgery”, “return rate”, “utilization rate”, “pregnancy”, and “live birth”. Controlled vocabulary and free-text terms were used where available. The final update also screened recent studies addressing phenotype-specific ovarian response, safety of oocyte retrieval in the presence of endometriomas, and emerging fertility-sparing approaches.

Original human studies reporting fertility-preservation procedures, ovarian stimulation outcomes, utilisation of cryopreserved material, or reproductive outcomes in women with endometriosis were prioritised. Guidelines, systematic reviews, and narrative reviews were used to contextualise the evidence and identify additional publications but were not included in the primary-study tables. Case reports, non-peer-reviewed publications, and studies without separately identifiable endometriosis data were excluded from the main evidence synthesis. Conference abstracts were not included in the primary-study tables; highly relevant recent prospective data not yet available as full-text publications were discussed only as preliminary contextual evidence and were explicitly identified as such.

Because the objective was to provide a broad and clinically oriented synthesis rather than answer a narrowly defined intervention question, this article was conducted as a comprehensive narrative review with a structured literature search and was not reported as a systematic review.

## 3. Endometriosis, Ovarian Reserve, and Reproductive Potential

### 3.1. Disease-Related Reproductive Impairment

The association between endometriosis and impaired fertility cannot be explained by a single mechanism. Depending on disease phenotype, reproductive potential may be affected by pelvic adhesions and altered tubo-ovarian relationships, chronic inflammation, changes in the follicular microenvironment, ovarian involvement, coexisting adenomyosis, and age-related decline occurring while pregnancy is postponed. The relative contribution of each mechanism differs between women and cannot be reliably inferred from disease stage alone [1,2].

Endometriosis-associated infertility should not, however, be equated with ovarian failure. Inflammatory changes, impaired tubo-ovarian anatomy, adhesions, and pain-related reduction in sexual activity may compromise natural conception without necessarily reducing the follicular pool. This distinction is clinically important for fertility-preservation counselling because oocyte cryopreservation may mitigate age- or ovary-related reproductive loss, but it does not correct tubal dysfunction, distorted pelvic anatomy, inflammatory mechanisms, or uterine factors [1,2].

Ovarian endometriomas are particularly relevant when fertility preservation is considered. They may be associated with reduced functional ovarian tissue, fibrosis of the surrounding cortex, and altered follicular recruitment. Women with ovarian endometriosis may consequently exhibit a lower oocyte yield during controlled ovarian stimulation. Recent phenotype-stratified ART data from 205 women found lower antral follicle count and fewer retrieved oocytes in ovarian endometriosis than in superficial or deep disease, reinforcing the importance of distinguishing ovarian involvement from other phenotypes; however, this was an IVF/ICSI cohort rather than a preventive fertility-preservation study [8]. The 2025 systematic review likewise found age and previous surgery to be more consistent predictors of fertility-preservation yield than lesion size or laterality [5].

Anti-Müllerian hormone (AMH) and antral follicle count (AFC) are useful for predicting the quantitative response to stimulation and for individualising gonadotrophin dosing, but neither independently predicts spontaneous fertility or establishes an indication for fertility preservation. A low AMH concentration may identify a woman likely to retrieve fewer oocytes, yet initiating fertility preservation only after ovarian reserve has markedly declined may substantially limit the number that can be stored.

### 3.2. Coexisting Adenomyosis and the Limits of Gamete Preservation

Adenomyosis frequently coexists with endometriosis and introduces a reproductive mechanism that is not directly addressed by oocyte cryopreservation. Preliminary results from the prospective multicentre Adenomyosis and Pregnancy Outcomes in Women Undergoing Assisted Reproductive Technology (AdAPT-ART) cohort included 813 women undergoing assisted reproductive technology, of whom 363 had ultrasound-diagnosed adenomyosis. Compared with women without adenomyosis, affected patients had lower adjusted probabilities of pregnancy, clinical pregnancy and live birth, together with higher risks of miscarriage and pregnancy loss. Notably, the reduction in live birth and the increased miscarriage risk persisted in donor-oocyte cycles and after euploid embryo transfer, supporting the hypothesis that adenomyosis may exert an intrinsic uterine effect independent, at least in part, of gamete or embryo competence [9].

The phenotypes most strongly associated with adverse outcomes included junctional-zone involvement, irregular or interrupted junctional zone, moderate-to-severe cavity distortion, involvement of more than 50% of the myometrium and more severe disease classifications. These findings reinforce the importance of phenotype-specific counselling. In women with coexisting endometriosis and adenomyosis, oocyte banking may preserve age-related gamete competence or protect against future ovarian loss, but it cannot preserve uterine receptivity or eliminate the risks associated with adenomyosis.

The reproductive consequences of adenomyosis may also extend beyond implantation and early pregnancy. In a cohort of 206 pregnant women with endometriosis, Scala et al. found that concomitant diffuse adenomyosis, but not focal adenomyosis, was associated with impaired placental markers and a substantially higher incidence of small-for-gestational-age birth (40.0% vs. 10.8% in women with endometriosis alone). Diffuse adenomyosis remained independently associated with small-for-gestational-age birth after adjustment for potential confounders (OR 3.744, 95% CI 1.158–12.099). These data further support a phenotype-specific uterine contribution that persists after conception and cannot be addressed by gamete cryopreservation alone [10].

### 3.3. Impact of Ovarian Surgery

Ovarian surgery constitutes one of the most consistent potentially modifiable factors associated with reduced oocyte yield. Damage may result from removal of healthy cortex adherent to the cyst wall, thermal injury during haemostasis, disruption of ovarian vascularisation, and repeated interventions for recurrent disease.

In the large cohort reported by Cobo et al., women with previous unilateral or bilateral endometrioma surgery had fewer retrieved and vitrified oocytes than women without previous surgery. Among women aged 35 years or younger, non-operated patients vitrified a mean of 8.6 mature oocytes per cycle compared with 5.1 after previous surgery, and cumulative live-birth rates were 72.5% and 52.8%, respectively [11].

Santulli et al. reached a similar conclusion in a prospective cohort. Previous surgery for ovarian endometriosis remained independently associated with a reduction in the number of oocytes retrieved after the first stimulation cycle after adjustment for age, AMH, gravidity, and gonadotrophin exposure [12]. Elizur et al. subsequently reported an adjusted 51.7% reduction in the number of mature oocytes among previously operated women [13].

Prospective evidence on ovarian reserve provides additional support for this association. Goodman et al. followed 116 women undergoing surgery for suspected endometriosis or endometriomas and found significantly lower baseline AMH concentrations in women with endometriomas than in women with a negative laparoscopy. Among women undergoing endometrioma excision, AMH declined by 48% overall at 1 month. In the subgroup analysis, the decline was significantly greater after bilateral than unilateral endometrioma surgery (53.0% vs. 17.5%). At 6 months, AMH remained below baseline but the difference was no longer statistically significant, suggesting that at least part of the early postoperative decline may recover over time [14].

The bilateral setting deserves particular attention because both ovaries are exposed to disease- and surgery-related loss of functional tissue. In Cobo et al., the mean number of mature oocytes available per patient was 10.3 ± 7.8 in women without previous ovarian surgery, 8.5 ± 4.8 after unilateral surgery, and 8.0 ± 5.7 after bilateral surgery [11]. Kim et al. reported 4.1 ± 2.9 cryopreserved oocytes in women with bilateral endometriomas compared with 5.7 ± 3.4 in those with unilateral disease, although this difference was not statistically significant in their small cohort [15]. Together with the greater postoperative AMH decline observed after bilateral surgery, these findings support fertility-preservation counselling before planned bilateral ovarian surgery. However, no comparative study has yet quantified the incremental live-birth benefit specifically attributable to fertility preservation before bilateral surgery.

These studies must be interpreted cautiously because none randomised women to surgery before or after fertility preservation. Operated and non-operated patients may differ in baseline ovarian reserve, disease severity, symptoms, and the indication for treatment. Preoperative AMH values were frequently unavailable. The available evidence therefore demonstrates a consistent association between previous surgery and lower ovarian response, but it cannot precisely quantify the causal effect for an individual patient.

Endometrioma size alone should not automatically determine the sequence of surgery and fertility preservation. Among non-operated women in the Elizur cohort, endometriomas larger than 4 cm were associated with similar AMH concentrations, numbers of retrieved oocytes, and mature-oocyte yields compared with smaller lesions [13]. Bilateral disease, recurrent endometriomas, and anticipated repeat surgery remain clinically concerning because they may expose a greater proportion of the follicular pool to disease- or treatment-related damage.

## 4. Fertility-Preservation Strategies

### 4.1. Oocyte Cryopreservation

Oocyte cryopreservation is the most extensively studied fertility-preservation strategy in women with endometriosis. All primary studies included in the recent systematic review used vitrification rather than slow freezing [5]. Its principal advantage is preservation of reproductive autonomy without requiring sperm from a partner or donor at the time of treatment.

The effectiveness of this strategy depends primarily on age at vitrification and the number of mature oocytes stored. In the Cobo cohort, younger women achieved higher oocyte survival, pregnancy, and cumulative live-birth rates than women older than 35 years [11]. A secondary analysis of the same cohort showed that cumulative live-birth probability increased with the number of oocytes used; however, upper estimates approaching 80–95% with approximately 20–22 oocytes were derived from small numbers of returners and were associated with wide confidence intervals [16]. These estimates should therefore not be interpreted as guaranteed individual probabilities.

Oocyte cryopreservation should therefore be presented as an opportunity to retain an additional reproductive option rather than as insurance against infertility. The ESHRE guideline considers the procedure feasible but notes that cost-effectiveness, patient-selection criteria, and true incremental benefit remain unknown [7].

The available evidence also indicates that feasibility should not be equated with proven population-level effectiveness. Although Cobo et al. reported an oocyte survival rate of 83.2% and a cumulative live-birth rate of 46.4% among women who returned to use their oocytes [11], Goldstein et al. found a cumulative utilisation rate of only 18% at 5 years in the overall cohort and 48% among women who actually attempted conception. Half of the pregnancies in that study were spontaneous [6].

### 4.2. Embryo Cryopreservation

Embryo cryopreservation is an established fertility-preservation option in assisted reproduction [17]. In endometriosis-specific cohorts, however, most women selected oocyte vitrification, with embryo cryopreservation used by a minority or in combination with oocytes. It may be considered when a patient has a stable reproductive partner or elects to use donor sperm and accepts the legal and personal implications of embryo storage.

Goldstein et al. reported that 32 of 200 women cryopreserved both oocytes and embryos, while no participant cryopreserved embryos alone. The presence of stored embryos was strongly associated with subsequent utilisation among women attempting conception, although this probably reflects a more immediate reproductive project rather than intrinsic superiority of embryo over oocyte cryopreservation [6].

### 4.3. Ovarian Tissue Cryopreservation

Ovarian tissue cryopreservation is an established fertility-preservation technique in selected settings, particularly when urgent gonadotoxic treatment does not allow time for controlled ovarian stimulation [17]. Evidence supporting its use specifically for endometriosis is extremely limited.

The procedure requires surgical removal of ovarian cortical tissue, whereas a central objective in endometriosis is often to avoid additional loss of functional ovarian tissue. Current cohorts do not provide disease-specific data on tissue transplantation, restoration of ovarian function, pregnancy, or live birth. It should therefore not be considered a routine first-line strategy for endometriosis and may be discussed only in highly selected circumstances or when another established medical indication coexists.

### 4.4. Emerging and Investigational Approaches

Minimally invasive treatment of endometriomas during a fertility-preservation pathway is being explored as a strategy to avoid or defer ovarian surgery. A 2026 prospective pilot study evaluated ethanol sclerotherapy performed during oocyte retrieval in 23 women with unilateral endometriomas seeking fertility preservation. The approach was feasible and was associated with substantial reduction in cyst volume, but the small sample and limited follow-up preclude conclusions regarding long-term reproductive benefit or routine clinical use [18]. Such approaches should currently be regarded as investigational and should not replace established counselling on the uncertainties surrounding surgery, expectant management, and oocyte banking.

## 5. Controlled Ovarian Stimulation

### 5.1. Pre-Stimulation Assessment

Before controlled ovarian stimulation, women should undergo assessment of age, ovarian reserve, pelvic anatomy, previous ovarian surgery, and the anticipated timing of any surgical procedure. AMH and AFC help estimate expected quantitative response but should not be equated with the probability of spontaneous conception or future live birth.

Previous ovarian surgery is among the most consistent predictors of a reduced response. Women who have already undergone surgery should not be excluded, but counselling should address the possibility of lower yield, repeated cycles, and diminishing benefit with increasing age.

### 5.2. Choice of Stimulation Protocol

Published studies have used heterogeneous ovarian stimulation protocols. Gonadotropin-releasing hormone (GnRH)-antagonist protocols were the most frequently employed, while other cohorts used short or long agonist protocols. Mathieu d’Argent et al. directly compared a GnRH-antagonist protocol with progestin-primed ovarian stimulation (PPOS) and found comparable numbers of retrieved and vitrified oocytes [19].

More recently, Calero et al. compared dienogest-based PPOS with antagonist and agonist protocols in a retrospective multicentre non-inferiority study. The dienogest protocol was non-inferior for mature-oocyte yield, without relevant differences in other ovarian response parameters, tolerance, or complications [20]. No comparative study has shown that one protocol provides superior cumulative live-birth outcomes in women with endometriosis. Comparative stimulation studies are summarised in Table 1.

**Table 1:** Comparative studies of controlled ovarian stimulation protocols.

Study	Protocols	Main Findings	Interpretation
Mathieu d’Argent et al., 2020 [19]	PPOS vs. GnRH antagonist	Similar retrieved and vitrified oocyte numbers; approximately 6.4 vitrified per woman	PPOS feasible; no live-birth follow-up
Calero et al., 2025 [20]	Dienogest-based PPOS vs. antagonist and agonist	Non-inferior mature-oocyte yield; similar tolerance and complications	Supports flexibility; retrospective allocation; no long-term reproductive outcomes

Abbreviations: PPOS, progestin-primed ovarian stimulation; GnRH, gonadotropin-releasing hormone.

### 5.3. Repeated Stimulation and Oocyte Accumulation

A single cycle may not provide the number of mature oocytes considered appropriate for the patient’s age and reproductive objectives. Repeated cycles were common in the principal cohorts. In the Santulli study, 82 of 146 women underwent more than one cycle, increasing mean cumulative oocyte yield from 8.4 after the first cycle to 13.6 per woman [12]. Kim et al. and Hong et al. similarly reported increasing cumulative yield with subsequent cycles [15,21].

The decision to repeat stimulation should be individualised according to age, first-cycle response, number already stored, AMH and AFC, treatment burden, cost, symptoms, surgical urgency, and desired family size. A fixed target applicable to all patients is not supported by current evidence.

### 5.4. Safety

Available observational data are generally reassuring regarding controlled ovarian stimulation, and a systematic review found no convincing evidence that ovarian stimulation increases endometriosis recurrence or progression [22].

In a recent single-centre cohort of 1668 oocyte-retrieval procedures in women with ovarian endometriomas, including 566 procedures performed for fertility preservation, endometrioma infection requiring surgical drainage occurred after 0.36% of procedures; no sepsis or septic shock was reported [23]. These data are reassuring but were obtained under standardised aseptic conditions with antibiotic prophylaxis, and therefore do not establish a universal prophylactic regimen.

Elizur et al. likewise reported no hospitalisation for pelvic inflammatory disease or tubo-ovarian abscess among 71 women undergoing 138 fertility-preservation cycles, including women with large and bilateral endometriomas [13]. Careful ultrasound planning, avoidance of endometrioma puncture when feasible, and individualised assessment of infection risk remain appropriate.

## 6. Reproductive Outcomes After Fertility Preservation

### 6.1. Laboratory and Post-Warming Outcomes

Evidence on laboratory and reproductive outcomes after warming remains concentrated in a small number of cohorts. Cobo et al. reported an overall oocyte survival rate of 83.2% after warming [11]. Successful warming did not invariably result in embryo transfer because attrition occurred through fertilisation failure, absence of viable or euploid embryos, embryo arrest, deferred transfer, and complete survival failure.

Previous surgery was associated with fewer retrieved and mature oocytes but not with lower maturation rates in the Elizur cohort [13]. The Cobo analyses similarly found no major difference in post-warming survival between non-operated, unilaterally operated, and bilaterally operated women [11]. This suggests that surgery may primarily reduce quantity rather than the competence of the oocytes successfully obtained, although available studies were not designed to establish that oocyte quality is unaffected.

### 6.2. Return and Utilisation

Return and utilisation rates are essential but strongly dependent on age at preservation, follow-up duration, relationship status, previous infertility, and the initial indication for treatment. Earlier studies reported highly variable return rates, reflecting differences in age and clinical context [5,11,12].

The short mean storage interval in the Cobo cohort suggests that many participants used cryopreservation as part of a short-term infertility-management pathway rather than conventional long-term preventive preservation [5,7]. This distinction supports separating “long-term fertility preservation” in women without an immediate pregnancy intention from “short-term fertility preservation” used as an adjunct before surgery or infertility treatment [12].

Goldstein et al. provided more informative longitudinal data: cumulative utilisation in the entire cohort was estimated at 7% at 2 years and 18% at 5 years, while among women who actually attempted conception it reached 48% at 5 years [6]. These 5-year estimates should be regarded as interim rather than definitive lifetime utilisation rates, because many women may not yet have reached their intended reproductive horizon; longer follow-up may therefore yield higher cumulative use of stored material.

### 6.3. Pregnancy and Live Birth

Cobo et al. reported 225 liveborn infants among the 485 women who returned to use their vitrified oocytes, corresponding to a cumulative live-birth rate of 46.4% among returners [11]. This should not be interpreted as a 46.4% probability for every woman initially undergoing fertility preservation because the analysis was restricted to women who returned.

Age markedly influenced outcome. Women aged 35 years or younger achieved a cumulative live-birth rate of 61.9%, compared with 28.4% in women older than 35 years [11]. A secondary Kaplan–Meier analysis estimated that cumulative live-birth probability increased with the number of oocytes used and was consistently higher at younger ages [16]. However, the upper-end projections approaching 80–95% were based on progressively smaller numbers of returners and were accompanied by wide and imprecise confidence intervals; they should therefore not be interpreted as individual probabilities or guaranteed outcomes.

Goldstein et al. evaluated women who attempted conception irrespective of method. Of 76 women, 48 achieved at least one pregnancy and 31 at least one live birth. Half of the pregnancies were natural, 37.5% followed use of cryopreserved oocytes or embryos, and 12.5% followed a new fresh in vitro fertilisation (IVF) attempt. Among 40 users of frozen material, 19 achieved pregnancy and 10 a live birth [6].

The Cobo and Goldstein studies therefore answer different questions. The former primarily describes outcomes among women returning to use vitrified oocytes; the latter better reflects the real-life reproductive trajectory after fertility preservation. The main clinical studies reporting fertility-preservation outcomes are summarised in Table 2.

**Table 2:** Main clinical studies reporting fertility-preservation outcomes in women with endometriosis.

Study and Design	Population	Key Findings	Main Limitations
Raad et al., 2018; retrospective cohort [24]	49 women; 70 cycles; mean age 33.9 years	Mean 9.5 oocytes retrieved and 7.2 MII oocytes/cycle; feasibility established	Small cohort; no reproductive follow-up
Cobo et al., 2020; retrospective multicentre [11]	1044 women vitrified; 485 returned; 840 cycles	Oocyte survival 83.2%; CLBR 46.4% among returners; lower yield after surgery	Returner analysis; short storage interval; mixed preventive/infertility-treatment context
Cobo et al., 2021; secondary analysis [16]	Same 485 women as [11]	CLBR increased with oocytes used; higher estimates in women ≤35 years	Same cohort as [11] ; upper Kaplan–Meier estimates imprecise with wide CIs and small numbers at the curve tail
Kim et al., 2020; retrospective [15]	34 women; 50 cycles; planned surgery	Repeated cycles increased cumulative oocyte yield	Small sample; no utilisation or live-birth data
Mathieu d’Argent et al., 2020; prospective observational [19]	108 women; PPOS vs. antagonist	Comparable retrieved and vitrified oocyte numbers (~6.4 vitrified/woman)	Non-randomised; no reproductive follow-up
Santulli et al., 2021; prospective cohort [12]	146 women; 258 cycles; mean age 31.5 years	Previous ovarian surgery independently reduced first-cycle yield; repeated cycles increased cumulative yield	Very low return at publication; non-standardised indications
Hong et al., 2021; retrospective [21]	62 women; 95 cycles; oocyte or embryo cryopreservation	Cumulative stored material increased with repeated cycles	Small later-cycle groups; mixed oocyte/embryo outcomes; no long-term follow-up
Elizur et al., 2023; retrospective two-centre [13]	71 women; 138 cycles; median age 31 years	Prior surgery associated with 51.7% fewer MII oocytes; lesions >4 cm did not reduce yield in non-operated women	No preoperative AMH; retrospective; no warming/live-birth outcomes
Goldstein et al., 2026; retrospective bicentric [6]	200 women; conception-attempt follow-up in 184	41.3% attempted conception; 63.2% pregnancy and 40.8% live birth among attempters; 18% 5-year utilisation overall; half of pregnancies spontaneous	Oocytes and embryos combined; retrospective; limited power for age/oocyte-number analyses

Abbreviations: MII, metaphase II; CLBR, cumulative live-birth rate; PPOS, progestin-primed ovarian stimulation.

### 6.4. Interpreting Effectiveness

Current evidence establishes technical feasibility and shows that warmed oocytes can result in live birth. It does not establish the additional probability of completing a desired family that is attributable to preventive cryopreservation compared with expectant management and later fertility treatment.

Effectiveness should be separated into three levels: technical effectiveness (retrieval, vitrification, and warming), reproductive effectiveness among users (pregnancy and live birth after stored material is used), and population-level effectiveness (additional family completion attributable to having undergone fertility preservation). Evidence is reassuring at the first level, limited but encouraging at the second, and insufficient at the third. The different denominators and their clinical interpretation are summarised in Table 3.

**Table 3:** Interpretation of reproductive outcome measures.

Outcome	Denominator	What It Measures	Main Limitation
Return rate	All women undergoing FP	Proportion returning to use stored material	Highly dependent on follow-up and reproductive intention
Utilization rate	All women or pregnancy attempters	Actual use of stored oocytes/embryos	Denominator varies between studies
Oocyte survival	Oocytes warmed	Technical cryopreservation performance	Does not account for fertilization or embryo development
Live birth among users	Women using stored material	Clinical outcome after utilization	Excludes non-returners
Live birth among pregnancy attempters	Women attempting conception	Overall real-life fertility	Includes spontaneous and fresh-IVF conceptions
Incremental benefit	Comparable women with vs. without FP	Benefit attributable to the intervention	Not yet established in endometriosis

Abbreviations: FP, fertility preservation; IVF, in vitro fertilisation.

### 6.5. Terminology: Preserving Gametes Is Not the Same as Preserving Fertility

The term “fertility preservation” is established in guidelines and clinical practice, but it may inadvertently imply that a future reproductive outcome has been secured. Capodanno et al. emphasised that high oocyte-survival and fertilisation rates are biological or laboratory outcomes and should not be conflated with the clinical objective of a live birth; they proposed more descriptive expressions such as “oocyte banking” or “gamete preservation” when appropriate [25].

Within endometriosis care, the distinction is clinically useful: cryopreserved oocytes preserve an additional reproductive resource, while age at use, oocyte survival, fertilisation, embryo development, uterine and tubal factors, coexisting adenomyosis, partner-related factors, disease progression, and the possibility of spontaneous conception continue to influence the eventual outcome. For consistency with international guidance, this review retains the conventional term “fertility preservation”, while recommending explicit counselling that the procedure preserves gametes and an option for future treatment rather than fertility itself.

## 7. Patient Selection and Optimal Timing

### 7.1. Early Counselling with Selective Intervention

Patient selection remains the most unresolved aspect of fertility preservation in endometriosis. A diagnosis of endometriosis alone should not be regarded as an automatic indication for oocyte cryopreservation. The distinction between counselling and treatment is essential: counselling provides information about risk and options, whereas treatment requires a judgement that stimulation and cryopreservation are proportionate to the anticipated risk, burden, and expected probability of benefit.

The 2022 ESHRE guideline strongly recommends counselling and information provision in extensive ovarian endometriosis, while explicitly stating that the true benefit remains unknown and that no criteria reliably identify women who will benefit [7].

### 7.2. Factors Relevant to Selection

Age at vitrification and anticipated delay before pregnancy.

AMH and AFC as predictors of response, not stand-alone indications.

Previous ovarian surgery and the likelihood of repeat surgery.

Bilateral or recurrent ovarian disease and limited residual healthy ovarian tissue.

Reproductive history, desired family size, and immediate versus deferred pregnancy plans.

Expected treatment burden, cost, access, and willingness to undergo repeated cycles.

Age should be considered both as a risk factor for future decline and as a determinant of treatment effectiveness. No single age threshold establishes eligibility, although published outcomes are consistently more favourable at 35 years or younger [11,16].

Endometrioma size alone is insufficient to determine whether treatment should be performed. Large lesions may affect access, symptoms, or surgical planning, but non-operated endometriomas larger than 4 cm did not reduce oocyte yield in the Elizur cohort [13].

Previous surgery, bilateral disease, recurrent endometriomas, and anticipated repeat surgery are clinically important because they may expose a larger proportion of the follicular pool to damage. The available evidence supports offering reproductive assessment before planned ovarian surgery whenever clinically feasible. Factors that may inform individualised counselling are summarised in Table 4.

**Table 4:** Factors to consider during fertility-preservation counselling.

Factor	Potential Implication	Clinical Interpretation
Younger age	Greater oocyte competence and yield	Earlier treatment is more efficient, but universal treatment risks overtreatment
Age > 35 years	Greater age-related risk but reduced efficiency	Individualize; avoid unnecessary delay
Low AMH/AFC	Lower expected yield	Response predictor, not stand-alone indication
Previous ovarian surgery	Consistently lower retrieved-oocyte yield	Supports counselling before further surgery
Bilateral/recurrent disease	Greater exposure of ovarian reserve to disease and treatment	Reasonable trigger for specialist referral
Large endometrioma	May affect access, symptoms, or surgical planning	Size alone does not mandate surgery or cryopreservation
Immediate pregnancy desire	Cryopreservation may delay direct conception attempts	Consider spontaneous conception or indicated infertility treatment
Long delay before pregnancy	Greater exposure to ageing and future intervention	Strengthens potential rationale
Desire for multiple children	Higher future oocyte requirement	Include in target planning and counselling

Abbreviations: AMH, anti-Müllerian hormone; AFC, antral follicle count.

### 7.3. Timing Relative to Surgery

The observational evidence consistently favours discussing fertility preservation before ovarian surgery rather than waiting for postoperative evidence of diminished reserve [11,12,13]. Fertility preservation before surgery is most defensible when surgery is elective, follicular access remains technically possible, and a brief delay will not compromise patient safety or symptom management.

Surgery may need to precede stimulation when there is suspicion of malignancy, severe uncontrolled symptoms, acute complications, or anatomy that makes retrieval unsafe or impossible. Fertility preservation after surgery remains possible but counselling should be realistic about expected yield.

In women not currently requiring surgery, the decision is less clear and should integrate age, bilateral involvement, disease evolution, ovarian reserve, expected delay to pregnancy, and patient preference. Current evidence does not support immediate cryopreservation for all women with stable non-operated endometriomas.

## 8. Counselling and Shared Decision-Making

Counselling should support an informed and preference-sensitive decision rather than direct every patient towards treatment. It should distinguish the risk of future reproductive impairment from the expected effectiveness of cryopreservation at the woman’s current age and ovarian reserve. Disease extent, rASRM stage, or clinical severity alone should not be considered an indication for fertility preservation in the absence of relevant ovarian involvement, planned ovarian surgery, age-related reproductive delay, declining ovarian reserve, or other reproductive risk factors [7,8].

Information should cover controlled ovarian stimulation, monitoring, retrieval, vitrification, long-term storage, warming, intracytoplasmic sperm injection, embryo development, and transfer. The possibility of requiring more than one cycle should be discussed before treatment.

Success estimates must identify the denominator used. Oocyte survival, pregnancy per transfer, live birth among material users, and live birth among all women undergoing fertility preservation are not interchangeable. Patients should understand that stored oocytes may never be used, not all warmed oocytes survive or fertilise, spontaneous conception may still occur, and cryopreservation does not eliminate the effects of endometriosis, adenomyosis, ageing, or other infertility factors.

In women with coexisting adenomyosis, counselling should explicitly separate preservation of gamete competence from future uterine reproductive potential, as adverse outcomes have also been reported after donor-oocyte and euploid embryo transfers [9].

Uncertainty should be communicated explicitly. Oocyte cryopreservation may preserve an additional reproductive option, particularly before potentially damaging ovarian surgery, but current evidence cannot determine whether an individual woman will need or ultimately benefit from the stored oocytes [25].

The choice between oocyte and embryo cryopreservation should reflect reproductive autonomy, sperm-source preferences, relationship status, legal considerations, and centre-specific rules. Psychological distress, anticipated infertility, treatment burden, and possible disappointment after a low yield should also be addressed.

Endometriosis-specific cost-effectiveness data are absent. Access may depend on reimbursement, employment flexibility, travel, and ability to finance repeated stimulation and storage. These factors should be discussed transparently without being confused with clinical risk.

**Figure 1:**
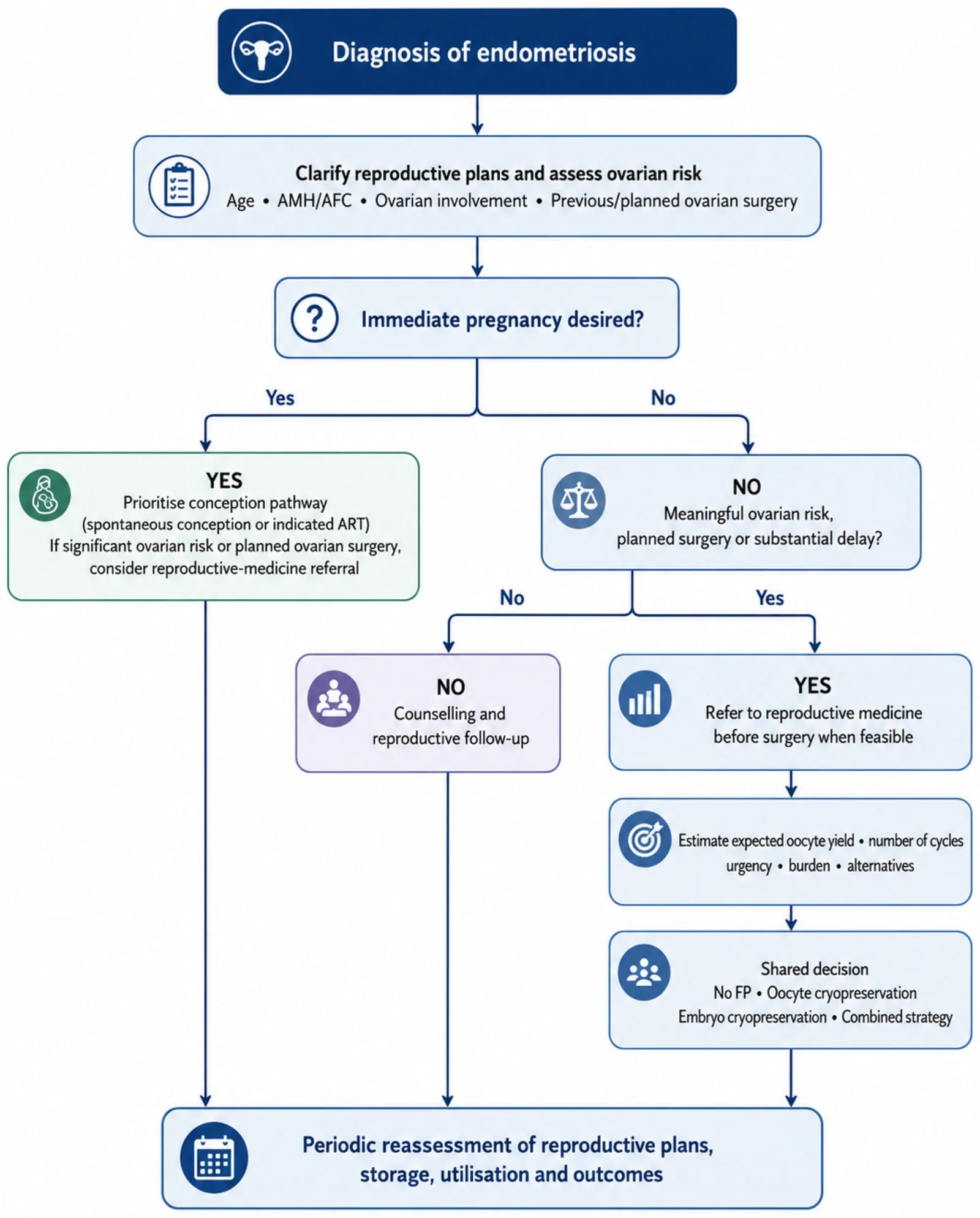
Proposed clinical pathway for fertility-preservation counselling in women with endometriosis. Note: This pathway is a proposed clinical framework derived from the available evidence and should not be interpreted as a validated prediction model or formal guideline.

A proposed clinical pathway integrating reproductive plans, ovarian risk, surgical timing and shared decision-making is shown in Figure 1.

## 9. Current Guidelines and Clinical Interpretation

The 2022 ESHRE endometriosis guideline provides the most directly applicable recommendation: clinicians should discuss the advantages and disadvantages of fertility preservation in women with extensive ovarian endometriosis, while acknowledging that the true benefit remains unknown [7]. The guideline notes the absence of validated selection criteria, uncertain cost-effectiveness, and limited evidence on ovarian tissue cryopreservation.

The 2020 ESHRE female fertility-preservation guideline provides general principles applicable to benign disease, including individualised information, assessment, established cryopreservation options, psychological support, and follow-up [17]. It does not define endometriosis-specific criteria for routine treatment.

French clinical practice recommendations developed through a modified Delphi process support informing women before surgery that it may damage ovarian reserve and about selectively considering oocyte vitrification in benign gynaecological disease [26].

No current guideline recommends routine oocyte cryopreservation for every woman with endometriosis. The areas of greatest agreement are early reproductive counselling, discussion before ovarian surgery, age-specific assessment of expected response and outcome, attention to bilateral or recurrent disease, and individualised shared decision-making.

## 10. Limitations of the Evidence and Future Research

The evidence remains limited in quantity and methodological quality. The recent systematic review identified only 10 observational studies, considerable heterogeneity, and no randomised trials [5]. Most cohorts were retrospective, single-centre or bicentric, and relatively small. Detailed reproductive outcomes in the largest dataset were restricted to women who returned, while the later “number needed to freeze” analysis used the same cohort and is not independent confirmatory evidence.

Several recent narrative and critical reviews have addressed fertility preservation in endometriosis from complementary perspectives, including available cryopreservation techniques and candidate selection, the balance between surgery and assisted reproduction, and the ovarian-reserve consequences of surgical treatment and emerging tissue-sparing approaches [27,28,29]. The present review builds on these syntheses by integrating the 2025 systematic evidence base with newer 2026 outcome and safety data, explicitly separating technical effectiveness, reproductive effectiveness among users, and population-level incremental benefit, and by incorporating phenotype-specific ovarian risk, adenomyosis-related uterine limitations, and patient-centred counselling.

Selection bias is substantial. Women referred for fertility preservation may differ from the broader endometriosis population in age, disease severity, socioeconomic resources, reproductive intentions, and access to specialist centres. Indications also varied between preventive long-term preservation and short-term adjunctive use before surgery or infertility treatment.

Comparisons between operated and non-operated women are confounded by baseline reserve, symptoms, severity, and treatment indication. Disease characterisation was inconsistent, and outcome denominators varied between cycles, women treated, women returning, warming procedures, and embryo transfers.

Future research should prioritise prospective multicentre registries with standardised baseline variables, clear separation of preventive and short-term strategies, and long-term follow-up through completed family size. Core outcomes should include cancellation, retrieved and mature oocytes, material stored, warming survival, fertilisation, blastocyst formation, pregnancy by conception method, utilisation, live birth, family completion, remaining material, costs, decisional conflict, treatment regret, and endometriosis-related health-related quality of life (HRQoL). Evaluating how fertility-preservation pathways affect HRQoL would complement reproductive endpoints and better capture treatment burden and patient-perceived benefit.

The most important unanswered question is comparative effectiveness. Randomised trials may be difficult, but carefully designed prospective cohorts using appropriate causal-inference methods could compare fertility preservation before surgery with surgery alone, preventive cryopreservation with expectant management, one versus repeated cycles, and oocyte versus combined oocyte–embryo strategies.

Validated patient-selection models should estimate both the probability that reproductive potential will be impaired before desired pregnancy and the probability of live birth from cryopreserved material if used. Endometriosis-specific economic evaluations and patient-reported outcomes are also required.

## 11. Conclusions

Endometriosis may compromise reproductive potential through ovarian involvement, inflammatory and anatomical mechanisms, ageing during delayed childbearing, and the effects of ovarian surgery. Oocyte vitrification is the most established and extensively studied fertility-preservation technique in this population, whereas embryo cryopreservation may be considered according to individual circumstances and ovarian tissue cryopreservation currently has a limited disease-specific role.

Available studies consistently indicate that increasing age and previous ovarian surgery are associated with lower oocyte yield. Recent phenotype-stratified evidence further supports distinguishing ovarian endometriosis from superficial or deep disease without ovarian involvement. These findings favour discussing reproductive plans and fertility preservation before ovarian surgery whenever clinically feasible.

Vitrified oocytes from women with endometriosis can survive warming and result in live birth. Age at cryopreservation and the number of mature oocytes available are major determinants of success. Recent follow-up data also demonstrate that many women conceive spontaneously and that utilisation depends strongly on reproductive intentions and duration of follow-up. Consequently, live births occurring after fertility preservation cannot automatically be attributed to the intervention.

Coexisting adenomyosis illustrates an important limitation of gamete-centred strategies: oocyte banking may preserve gamete competence while uterine factors continue to influence implantation, miscarriage, placentation and pregnancy outcomes.

Current evidence does not support routine oocyte cryopreservation for every woman with endometriosis. The most appropriate strategy is early counselling combined with selective, individualised treatment. Particular attention should be given to women with bilateral or recurrent ovarian disease, previous or planned ovarian surgery, delayed reproductive plans, or a desire for multiple future children.

Patients should understand that oocyte banking preserves reproductive material and an additional possibility rather than fertility itself or a guaranteed future live birth. Until comparative long-term data are available, multidisciplinary counselling and shared decision-making remain the cornerstone of care.

## Data Availability

No new data were created or analyzed in this study. Data sharing is not applicable to this article.
